# Synergistic effect of garlic and andrographis extracts on digestive enzyme activity, immune modulation, and anti- inflammation in nile tilapia (*Oreochromis niloticus*)

**DOI:** 10.1016/j.cirep.2025.200257

**Published:** 2025-10-22

**Authors:** Mallika Supa-aksorn, Sudaporn Tongsiri, Jongkon Promya, Chetsalit Hongnueng, Doungporn Amornlerdpison

**Affiliations:** a Faculty of Fisheries Technology and Aquatic Resources, Maejo University, Chiangmai, 50290, Thailand; b Center of Excellence in Agricultural Innovation for Graduate Entrepreneur, Maejo University, Chiangmai, , 50290Thailand

**Keywords:** Andrographis extract, Anti-inflammatory effect, Digestive enzymes, Immune, Nile tilapia, Garlic extract

## Abstract

This research investigated the effects and their mechanisms of garlic extract (GE), Andrographis extract (AE), and their combined formulation (GAF) on digestion, immune response, and inflammation in Nile tilapia (*Oreochromis niloticus*), a freshwater species commonly raised in high-density aquaculture systems prone to stress and disease. Following 14 days of fish dietary supplementation, the GAF group showed significantly enhanced digestive enzyme activities, including amylase, lipase, and trypsin while chymotrypsin was reduced, yielding a higher trypsin/chymotrypsin ratio indicative of improved protein digestion. The GE and GAF groups demonstrated a positive relationship between a higher T/C ratio and a trend toward improved growth performance. Lysozyme activity, a key innate immune marker, was also significantly elevated in the GAF group, followed by AE and GE. *In vitro* tests using RAW 264.7 macrophages confirmed no cytotoxicity at extract concentrations of 250–4000 µg/mL, and increased cell viability at 1000–2000 µg/mL. Gene expression analysis demonstrated that AE strongly suppressed proinflammatory markers (IL-1β, TNF-α, iNOS, IL-6), with GAF and GE showing moderate effects. All treated groups significantly lowered the IL-6/IL-10 ratio, reflecting improved immune balance. Collectively, AE, GE, and GAF enhance digestive efficiency, boost immune function, and anti- inflammation. The balanced response from GAF supports its use as a natural feed additive to reduce antibiotic reliance and promote sustainable aquaculture.

## Highlights

• This study was designed to evaluate the biological effects of Allum sativum (GE), Andrographis paniculata (AE), and a combined 1:1 formulation (GAF) on digestive enzyme activities and immune responses in Nile tilapia at organismal level.
• The effects of these extracts were assessed in LPS-induced RAW 264.7 cells by analyzing cell viability and inflammatory cytokine gene expression, to provide mechanism of action on immune response at cellular levels.
• The GAF formulation exhibited a well-balanced effect on both the digestive and immune systems.

## Introduction

Aquaculture plays a pivotal role in global food security, particularly in the cultivation of Nile tilapia (*Oreochromis niloticus*), a freshwater species of high economic significance. This species is characterized by its rapid growth rate, broad environmental adaptability, and its ability to provide a high-quality protein source for human consumption ([1,2]. However, intensive aquaculture systems, which often involve high stocking densities, can lead to chronic stress in fish, resulting in immunosuppression, increased susceptibility to infectious diseases, and diminished digestive efficiency [3]. These challenges underscore the urgent need for safe and sustainable strategies to enhance both the immune system and digestive function in cultured aquatic animals.

Among various alternatives, the use of medicinal plant extracts as dietary supplements in aquaculture has received growing attention due to their rich content of bioactive compounds with antimicrobial, immunomodulatory, and digestive-enhancing properties [4]. Notably, *Andrographis paniculata* contains andrographolide, a diterpenoid compound with well-documented anti-inflammatory, immunostimulatory, and antiviral activities [5]. Similarly, *Allium sativum* (garlic) contains allicin, an organosulfur compound that exhibits antioxidant, antimicrobial, and digestive-promoting effects, as well as the ability to activate various immune cell types [6]. In addition, our previous study showed that *Allium sativum* (GE), *Andrographis paniculata* (AE), and a combined 1:1 formulation (GAF) against *Aeromonas hydrophila,* a pathogenic bacterial infection in Nile tilapia. Results demonstrated that the GAF formula exhibited the strongest inhibitory effect on *A. hydrophila*, followed by AE and GE, which showed comparable activity [7].

Several *in vivo* studies have demonstrated the beneficial effects of *Allium sativum* and *Andrographis paniculata* in aquatic species, particularly in enhancing immune response and disease resistance [4]. Therefore, this study further investigated these effects *in vitro* using RAW 264.7 cells, a murine macrophage cell line commonly employed as a model of innate immunity. These cells respond specifically to stimuli, such as lipopolysaccharide (LPS), (LPS) by secreting inflammatory cytokines including IL-1β, TNF-α, iNOS, IL-6, and IL-10 [8]. Assessing cytokine gene expression in RAW 264.7 cells provide a reliable model for evaluating the anti-inflammatory potential of natural compounds at the molecular level. Given that RAW 264.7 macrophages exhibit a strong and specific cytokine expression profile in response to LPS stimulation [8;9], any significant suppression of proinflammatory gene expression by plant extracts would indicate their potential as safe and effective immunomodulators, applicable both at the cellular and organismal levels [10].

Accordingly, this study was designed to evaluate the biological effects of *Allum sativum* (GE), *Andrographis paniculata* (AE), and a combined 1:1 formulation (GAF) on digestive enzyme activities, immune responses, and growth performance in Nile tilapia. In parallel, the effects of these extracts were assessed in LPS-induced RAW 264.7 cells by analyzing cell viability and inflammatory cytokine gene expression, to provide mechanism of action at both organismal and cellular levels that may extend beyond aquaculture and hold potential for improving health in terrestrial livestock systems.

## Materials and methods

### Preparation of herb extract

Garlic extract (GE), Andrographis extract (AE), and a 1:1 combination of the two (GAF) were prepared as follows: Garlic cloves were purchased from Mae Jo Market, San Sai District, Chiang Mai, Thailand, and Andrographis leaves were obtained from Thung Khao Phuang Subdistrict, Chiang Dao District, Chiang Mai, Thailand. The herbs were washed and oven-dried at 50 °C, then ground into fine powders. Each powder was macerated in ethanol at a 1:10 (w/v) ratio for three days. The mixtures were filtered through filter membranes, and the solvents were removed using a rotary evaporator under reduced pressure, following a modified method by Kaewphibool and Bunnak [11]. The ethanolic garlic extract was further dried using a spray dryer, based on a modified method from Poojitha et al [12]. Likewise, the Andrographis extract was dried using the same spray drying method, modified by Tongthong et al [13].

The percentage yield of the crude extract was calculated, and the extracts were stored at 4 °C until further experiment. The morphological characteristics and particle size of the extract-derived particles were investigated using Scanning Electron Microscopy (SEM).

### Fish rearing

A total of 240 juvenile Nile Tilapia (*Oreochromis niloticus*), with an initial weight of 10–30 g, were divided into four groups: (1) A control group fed with a commercial diet. (2) A group fed with a diet containing GE. (3) A group fed with a diet containing AE. (4) A group fed with a diet containing GAF at a concentration of 1.0 % (w/w). All groups were fed once a day at 5 % body weight for 14 days. Growth performance was evaluated based on weight gain (WG), percentage weight gain (%WG), and feed conversion ratio (FCR). WG was calculated as the difference between final and initial body weights, while %WG was determined as ((Final Weight – Initial Weight) / Initial Weight) × 100. FCR was calculated as the ratio of feed intake to weight gain. Fish were anesthetized with clove oil (250 mg/L), as confirmed by reduced activity, slower respiration, and a lack of response to external stimuli. Blood samples were collected from the caudal vein using sterile syringes for immunological analysis. The abdominal cavity was then dissected to collect small intestine samples, which were rinsed with sterile PBS and stored at −80 °C for further analysis. Digestive enzyme activities, including amylase, lipase, trypsin, and chymotrypsin were measured. The immunity was assessed through humoral immune responses using lysozyme analysis.

### Determinations of digestive enzymes

#### Enzymes extraction

Enzyme extraction was performed according to Rungruangsak-Torrissen [14]. Frozen digestive tracts were homogenized in 50 mM Tris–HCl buffer (pH 8) containing 200 mM NaCl at a 1:2 (w/v) ratio. The supernatant) was collected by centrifugation at 10,000 × *g* for 20 min at 4 °C. After that, the crude enzyme extracts were determined protein concentrations according to Lowry et al [15], using bovine serum albumin (BSA) as a standard.

#### Amylase-specific activity

Amylase activity was determined based on a modified method from Areekijseree et al [16], originally described by Bernfeld [17]. Reaction mixtures consisted of 25 µl of 5 % soluble starch, 62.5 µl of 0.2 M phosphate buffer (pH 7), 37.5 µl of 20 mM NaCl, and 125 µl of diluted crude enzyme extract. After incubation for 15 min at ambient temperature, 250 µl of 1 % 3,5-dinitrosalicylic acid solution was added, followed by boiling for 5 min. Then, 2.5 ml of distilled water was added. Absorbance at 540 nm was measured and compared to a maltose standard curve. The enzyme-specific activity was expressed as µmol maltose h^–1^ mg protein^–1^.

#### Lipase-specific activity

Lipase activity was analysed using p-nitrophenyl palmitate as a substrate, following the method of Winkler & Stuckmann [18]. Reaction mixtures contained 200 µl of 0.01 M p-nitrophenyl palmitate, 800 µl of 0.2 M phosphate buffer (pH 8), and 10 µl of crude enzyme extract. The reaction proceeded for 15 min at ambient temperature, then stopped with 250 µl of 1 M Na_2_CO_3_. After centrifugation at 10,000 × *g* for 15 min, absorbance at 410 nm was measured. Specific activity of lipase was expressed as µmol *p*-nitrophenol produced h^–1^ mg protein^–1^.

#### Trypsin and chymotrypsin-specific activities

Trypsin and chymotrypsin activity were determined using 1.25 mM benzoyl-*l*-arginine-*p*-nitroanilide (BAPNA) and 0.1 mM *N*-succinyl-Ala-Ala-Pro-Phe-*p*-nitroanilide (SAPNA), respectively, according to Rungruangsak-Torrissen [14]. Each substrate was dissolved in dimethylformamide (5 % final concentration) before making up to final volume with 0.2 M Tris buffer pH 9. The reaction mixture of 10 µl crude enzyme extract and 1000 µl substrate solution was incubated at ambient temperature, and increased absorbance of one-minute initial reaction rate was measured at 410 nm. Both trypsin and chymotrypsin specific activities were expressed as µmol *p*-nitroaniline produced h^–1^ mg protein^–1^. The digestive efficiency was expressed as the activity ratio of Trypsin to Chymotrypsin (T/C ratio), as described by Rungruangsak-Torrissen *et al* [19] and Rungruangsak-Torrissen [20].

### Non-specific immune assays

#### Serum lysozyme activity

Blood samples were collected in micro-centrifuge tubes, and stored overnight at 4 °C. Serum was obtained by centrifuging blood sample at 10,000 rpm for 10 min at 4 °C. Lysozyme activity in the serum was assayed by mixing with bacteria *Micrococcus lysodeikticus* suspension and measured spectrophotometrically at 540 nm, according to Puangkaew *et al* [21] modified from Ellis [22]. The results are given as units (U) ml^–1^ where one unit is the amount of sample causing a decrease in absorbance of 0.001 min^–1^.

### Anti-inflammatory effect and immune response in RAW 264.7 cells

#### Cell culture

The murine macrophage cell line (RAW 264.7, TIB-71, ATCC) was cultured using Dulbecco's Modified Eagle media (DMEM, Gibco 11,995–065, UK) supplemented with 10 % fetal bovine serum (FBS, Gibco, UK), and 1 % antibiotic–antimycotic solution (Gibco, USA). Briefly, the cells were treated at 37 °C in a humidified atmosphere of 5 % CO_2_.

#### Cell viability determination

Cell viability was assessed using the PrestoBlue® Assay following the manufacturer’s protocol. RAW 264.7 cells were seeded in 100 μl aliquots into a 96-well plate at a final density of 2 × 10⁵ viable cells per well. The cells were then cultured in DMEM supplemented with Garlic extract (GE), Andrographis extract (AE), and a combination of Garlic and Andrographis extracts in a 1:1 ratio (GAF) at concentrations (500 μg/ml) with 0.5 μg/ml lipopolysaccharide (LPS, Invitrogen, USA) for 24 h. The control group was maintained in media without extracts and. At the end of incubation period, the media was replaced with fresh media containing 1 % v/v PrestoBlue® (Thermo Fisher Scientific A13261) and incubated for 3 h at 37 °C. Cell viability was determined by measuring the absorbance of PrestoBlue® reduction using a microplate reader (SpectraMax iD5, Molecular Devices) at 570 nm with a reference wavelength of 600 nm.

#### Determination of cytokine gene expression by real-time PCR

Raw 264.7 cells were incubated in DMEM media supplemented with selected concentration of the sample (500 μg/ml) with 0.5 μg/ml LPS to induce the inflammatory response in the macrophages. The cells were incubated for 24 h. The control group was maintained in media without extracts and LPS and LPS group was maintained in media with 0.5 μg/ml LPS supplementation only. After incubation, the cells were collected for RNA extraction using Trizol® Reagent (Thermo Fisher Scientific 15,596,026, USA) then cDNA synthesis using RevertAid First Strand cDNA Synthesis Kit (Molecular Biology 3095,476, Lithuania) following the manufacturer’s protocol. The quantitative detection of inflammatory cytokine including inducible nitric oxide synthase (iNOS), tumor necrosis factor-alpha (TNF-α), Interleukin-1beta (IL-1β), Interleukin-6 (IL-6) and anti-inflammatory cytokine as Interleukin-10 (IL-10) were performed using real-time PCR (qPCR) SensiFAST™ SYBR® No-ROX Kit (Bioline, SFSN-424208A) was used. The sequence of primers shows in Table 1. The PCR protocol consisted of 40 cycles of denaturation at 95 °C for 15 s, annealing at 60 °C for 10 s followed by 72 °C for 15 s to allow for extension and amplification of the target sequence. Glyceraldehyde-3-Phosphate Dehydrogenase (GAPDH) was used as the endogenous housekeeping gene to normalize the expression of the targeted genes Table 2..

**Table 1:** Sequence of primers used for qPCR.

Gene Name	Gene Bank Number	Primer Sequence (5′ - 3′)	Product Size (bp)	Annealing Temperature ( °C)
iNOS	NM_010927.4	Forward CTGCAGCACTTGGATCAGGA	64	60
		Reverse GACACTTCGCACAAAGCAGG		
TNF-α	NM_013693.3	Forward AGCAAACCACCAAGTGGAGGA	105	60
		Reverse GCTGGCACCACTAGTTGGTTGT		
IL-1β	NM_008361.4	Forward AGTTGACGGACCCCAAAAG	75	60
		Reverse AGCTGGATGCTCTCATCAGG		
IL-6	NM_031168.2	Forward GCTACCAAA CTGGATATAATCAGGA	78	60
		Reverse CCAGGTAGCTATGGTACTCCAGAA		
IL-10	NM_010548.2	Forward CAGAGCCACATGCTCCTAGA	79	60
		Reverse TGTCCAGCTGGTCCTTTGTT		
GAPDH	NM_008084.4	Forward TCTCTGCTCCTCCCTGTTCC	71	60
		Reverse TTTTGTCTACGGGACGAGGC

**Table 2:** Trypsin/ Chymotrypsin ratio and Growth performance of Nile tilapia after 14 days of feeding.

Group	T/C ratio	WG ( %)	FCR
Control	0.783 ± 0.069 ^d^	51.23 ± 16.69^a^	1.51 ± 0.47 ^a^
AE	2.128 ± 0.156 ^c^	53.00 ± 17.33 ^a^	1.45 ± 0.43 ^a^
GE	3.729 ± 0.116 ^b^	57.28 ± 18.32 ^a^	1.35 ± 0.41 ^a^
GAF	5.169 ± 0.227 ^a^	57.56 ± 19.37 ^a^	1.34 ± 0.39 ^a^

Values are expressed as mean ± SD (n = 60 fish per group). Means in the same column with different superscripts differ significantly (p < 0.05).

#### Statistical analysis

The results for digestive enzymes, non-specific immune assays, and growth performance were expressed as mean ± standard deviation (SD). Multiple group comparisons were conducted using one-way analysis of variance (ANOVA), in cases of significant differences (*p* < 0.05). Statistical analyses were performed using the SPSS software version 29. For the experiment in RAW 264.7 cells, statistical significance was evaluated using one-way ANOVA, in cases of significant differences (*p* < 0.01). Statistical analyses were performed using the GraphPad Prism 9 software.

## Results

### Extracts and formulation

**Fig. 1:**
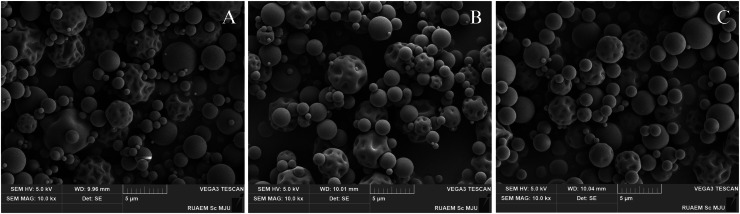
Scanning Electron Microscopy (SEM) images at 10,000 × magnification showing the physical morphology of particles derived from three herbal extract groups: A; Garlic extract (GE), B; Andrographis extract (AE), and C; a combined formulation of AE and GE (GAF).

The yields of GE and AE were found at 12 and 3 %, respectively, [7]. The SEM analysis revealed that the particles of these extracts exhibited generally spherical morphology and loose aggregation. The average particle sizes of the AE, GE, and GAF extracts were found to range between 619 and 689 nanometers in Fig. 1, indicating a broad size distribution. Notably, these average sizes exceed the ISO-defined threshold for nanoparticles (≤100 nm), and thus the materials are classified as micro-sized particles (non-nano particles). Despite their larger size, the relatively smooth surface texture and good dispersibility suggest potential suitability for applications in bioproduct formulations.

### Digestive enzymes

**Fig. 2:**
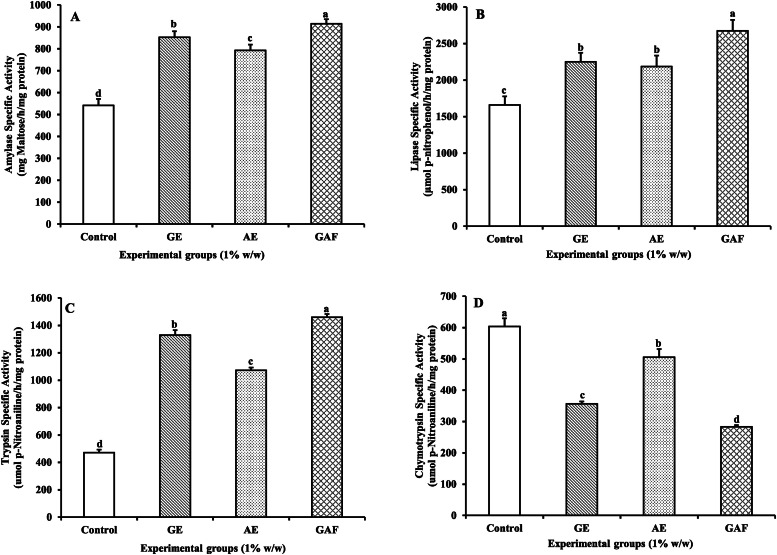
Specific enzyme activity (A), amylase activity (B), lipase activity (C), trypsin activity (D), and chymotrypsin activity of Nile tilapia fed with different diet formulations over a 14-day period.

The specific activity of the amylase enzyme showed the highest activity in the GAF group, with an average value of 914.18±20.43 mg Maltose/h/mg protein. This was followed by the GE group, with an average of 851.99±27.36 mg Maltose/h/mg protein. The AE group exhibited a slight decrease compared to the GE group but remained higher than the control group, with an average value of 792.86±26.49 mg Maltose/h/mg protein. The lowest activity was observed in the control group, with an average value of 541.73±29.10 mg Maltose/h/mg protein. The differences among the groups were statistically significant (*p* < 0.05), (Fig. 2A).

The specific activity of lipase enzyme showed the highest activity in the GAF group, with a value of 2674.38±148.10 µmol p-Nitrophenol/hr/mg protein, while the control group had the lowest value, at 1658.69±117.49 µmol p-Nitrophenol/hr/mg protein. This was statistically significant when compared to the GE and AE groups, which showed similar values of 2248.63±124.83 and 2186.60±148.52 µmol p-Nitrophenol/hr/mg protein, respectively, (Fig. 2B).

The specific activity of trypsin enzyme was the most significantly elevated in the GAF group, with a value of 1459.76±22.34 µmol p-Nitroaniline/hr/mg protein. The GE group followed with a value of 1328.28±36.94 µmol p-Nitroaniline/hr/mg protein. The AE group had a slightly lower value compared to the GE group, at 1070.85±21.22 µmol p-Nitroaniline/hr/mg protein, while the control group had the lowest value of 470.42±22.84 µmol p-Nitroaniline/hr/mg protein, (Fig. 2C). In contrast, the specific activity of chymotrypsin enzyme showed an inverse relationship with the trypsin enzyme activity. It showed the highest activity in the control group, with a value of 603.28±26.38 µmol p-Nitroaniline/hr/mg protein, and lowest in the GAF group, with a significantly lower value of 282.71±5.85 µmol p-Nitroaniline/hr/mg protein, (Fig. 2D).

As shown in Table 2, the trypsin/chymotrypsin (T/C) ratio of Nile tilapia increased significantly with dietary supplementation, with the highest values recorded in the GAF group, followed by GE, AE, and the lowest in the control group. This result clearly indicates that dietary supplementation enhanced digestive protease activity. Although %WG and FCR did not differ significantly among groups (*p* > 0.05), the observed trend suggests a positive relationship between higher T/C ratio and improved growth performance. Specifically, GE and GAF groups exhibited both higher T/C ratios and slightly higher %WG values, as well as lower FCR compared to the control. This implies that the T/C ratio may serve as a useful physiological indicator to predict growth potential and feed utilization efficiency in Nile tilapia.

### Non-specific immune assays

**Fig. 3:**
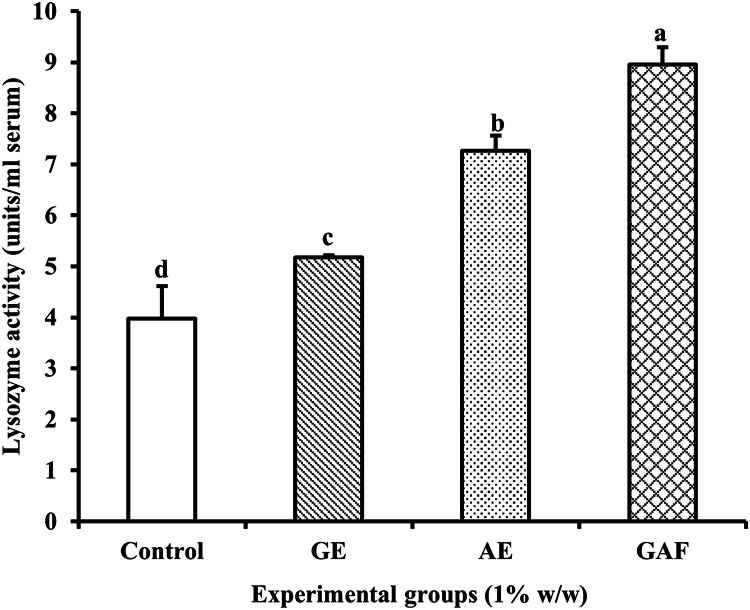
Lysozyme activity of Nile tilapia fed with different diet formulations over a 14-day period.

The lysozyme activity is significantly higher in the GAF group compared to the other experimental groups, followed by GE, AE, and Control, significant difference (*p* < 0.05). (Fig. 3).

### Cell viability

**Fig. 4:**
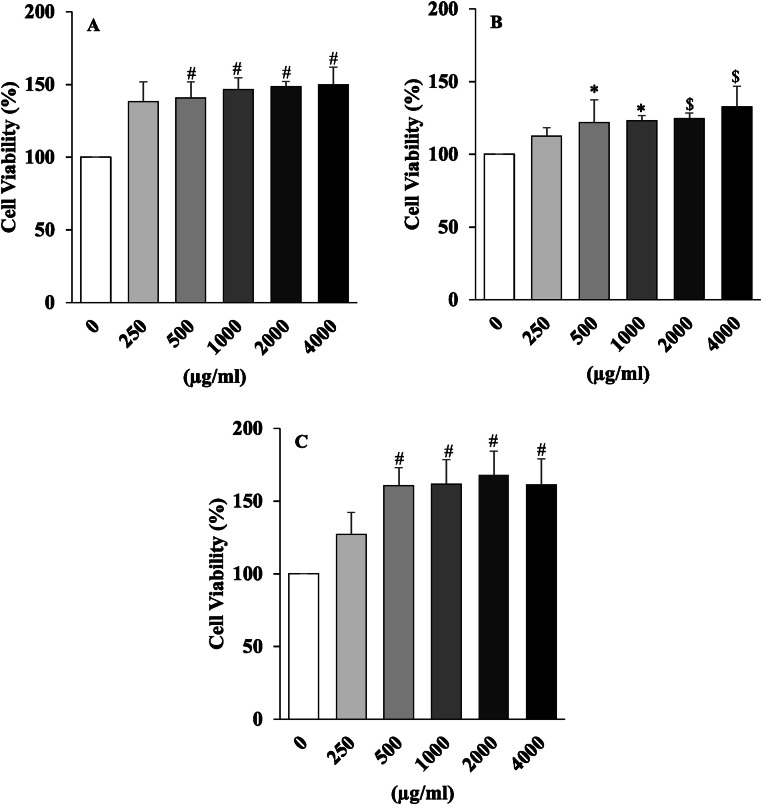
The effects of GE (A), AE (B), and GAF (C) on RAW 264.7 macrophage cells were assessed using the Presto Blue assay. Data are expressed as mean ± SEM and each experiment included 4 repeats per condition, with significance levels indicated as follows: * (p < 0.05), $ (p < 0.001), and # (p < 0.0001) when compare to control group, as determined by one-way ANOVA, (n = 4).

The effect of GE, AE, and GAF on the viability of RAW 264.7 macrophage cells were assessed using the PrestoBlue assay at concentrations of 250, 500, 1000, 2000, and 4000 μg/mL. The results are presented in Fig. 4. Treatment with AE resulted in a dose-dependent increase in cell viability across all tested concentrations. At 250 μg/mL, the viability of RAW 264.7 cells increased significantly to 112.36 ± 6.80 % compared to the control group (*p* < 0.05). Further increases were observed at 500–2000 μg/mL, with an average viability of 123.11 %. At the highest concentration (4000 μg/mL), AE-treated cells showed a significantly enhanced viability compared to the control (*p* < 0.01), suggesting that AE may promote macrophage metabolic activity at moderate to high doses. (Fig. 4A)

Similarly, GE extract treatment led to a progressive enhancement of cell viability in RAW 264.7 cells from 500 μg/mL to 4000 μg/mL. The increase in viability was statistically significant at all tested concentrations (*p* < 0.01), indicating that GE supports cell survival and may exert immunoenhancing effects with minimal cytotoxicity. (Fig. 4B)

The combined extract, GAF also demonstrated significant enhancement of cell viability. At 250 μg/mL, viability increased significantly (*p* < 0.05), while at 1000 and 2000 μg/mL, a highly significant increase was observed (*p* < 0.01). Notably, at 4000 μg/mL, cell viability peaked but did not show a statistically significant difference compared to 2000 μg/mL, suggesting a potential saturation point of stimulatory effect. (Fig. 4C)

### Effects of GE, AE, and GAE on cytokine expression

**Fig. 5:**
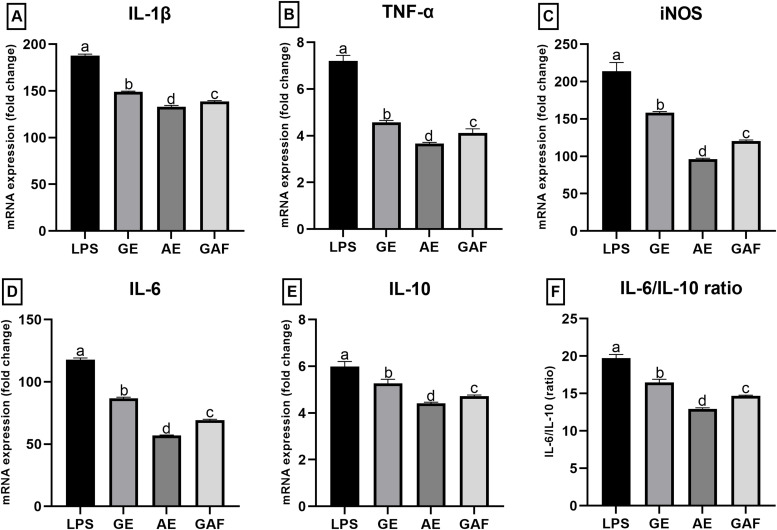
Effects of GE, AE, and GAF on cytokine including IL-1β (A), TNF-α (B), iNOS (C), IL-6 (D), IL-10 (E), and IL-6/IL-10 ratio (F) expression in LPS-induced Raw 264.7 macrophage cells. Values are presented as Mean ± SEM from three replicates per group. Means ± SEM with different letters is significantly different according to Tukey’s test (p < 0.05).

In this study, the effects of GE, AE, and GAF were evaluated on the expression of inflammation-related cytokines, including IL-1β, TNF-α, iNOS, IL-6, and IL-10, as well as the IL-6/IL-10 ratio. RAW 264.7 macrophage cells were stimulated with LPS (0.5 µg/mL) and treated with each extract at a concentration of 500 µg/mL for 24 h. The mRNA expression levels were analyzed using RT-qPCR. The results revealed that the expression of IL-1β was significantly downregulated in all treatment groups, with the most pronounced reduction observed in the AE-treated group compared to the LPS-only group (Fig. 5A). In the case of TNF-α, all three extracts effectively reduced gene expression, with AE exhibiting the strongest inhibitory effect; however, no statistically significant difference was found between the GE and GAF groups (Fig. 5B). Regarding iNOS, a key enzyme involved in nitric oxide production during inflammation, the expression was most notably suppressed by AE, followed by GE and GAF, respectively (Fig. 5C). IL-6 expression was decreased in all treatment groups relative to the LPS control, with AE showing the greatest level of suppression, consistent with the known anti-inflammatory properties of its active compounds (Fig. 5D). For IL-10, an anti-inflammatory cytokine, a downward trend in expression was observed in all extract-treated groups, particularly in the AE group; however, no significant difference was detected between GE and GAF (Fig. 5E). With respect to the IL-6/IL-10 ratio a marker of the balance between pro- and anti-inflammatory responses all extract groups significantly reduced this ratio, with AE again demonstrating the most significant reduction (Fig. 5F). Overall, these findings suggest that AE possesses the highest potential for suppressing the expression of inflammation-associated cytokine genes in LPS-stimulated RAW 264.7 cells. While GE and GAF also exhibited anti-inflammatory effects, their efficacy was comparatively lower than that of AE, with statistically significant differences observed for specific cytokine targets.

## Discussion

The findings of this study revealed that dietary supplementation with GE, Andrographis AE, and GAF over a 14-day period significantly enhanced the specific activities of digestive enzymes in Nile tilapia. Notably, the GAF group exhibited the highest activities of amylase, lipase, and trypsin, whereas the activity of chymotrypsin was significantly reduced. These results indicate that herbal extracts, especially in combination, may improve digestive function through multiple mechanisms including stimulation of enzyme secretion, modulation of gut microbiota, and regulation of oxidative stress [4,6]. Garlic is rich in sulfur-containing compounds such as allicin, S-allyl cysteine (SAC), and diallyl disulfide (DADS), which have been reported to stimulate bile secretion, enhance intestinal motility, and promote the release of digestive enzymes from the pancreas and gut mucosa [23,24].

In this study, the GE group showed significantly higher activities of amylase, lipase, and trypsin compared to the control. These effects may be attributed to allicin’s ability to activate enzyme secretion through cholinergic and nitric oxide-dependent pathways, which enhance mucosal response to nutrients [25]. Although *A. paniculata* is widely recognized for its anti-inflammatory and antiviral activities, its active compound andrographolide also may improve intestinal absorption by reducing gut inflammation and protecting epithelial integrity under oxidative stress [26,27]. In the current study, AE supplementation significantly enhanced amylase and trypsin activities, albeit to a slightly lesser extent than GE and GAF. This suggests that AE may exert indirect digestive benefits by preserving gut health and reducing inflammation, thereby supporting enzymatic function. The GAF exhibited the most pronounced effects across all enzymatic indices, except chymotrypsin, which was significantly decreased. This outcome is considered beneficial, as it indicates improved protein digestion primarily via trypsin. The synergy between allicin and andrographolide may involve both stimulation of enzyme secretion and attenuation of inflammation and oxidative damage, leading to enhanced digestive enzyme efficiency [5,27]. The observation of high trypsin activity and low chymotrypsin activity in the GAF group is particularly noteworthy, as the trypsin-to-chymotrypsin ratio (T/C ratio) is a reliable biomarker for assessing protein digestive efficiency in freshwater fish [[14], [28]]. This enzymatic balance may reflect enhanced nutrient assimilation, potentially contributing to improved growth performance and feed conversion efficiency in longer-term applications. The present findings also highlight the relationship between the T/C ratio and growth performance. Groups exhibiting higher T/C ratios (GE and GAF) tended to show improved %WG and reduced FCR, even though the differences were not statistically significant. This trend supports the notion that the T/C ratio can serve as a physiological predictor of digestive efficiency and subsequent growth performance in fish.

The lysozyme activity analysis revealed that GAF-treated fish exhibited the highest immune the results of this study revealed that dietary supplementation with GE, AE, and GAF over a 14-day period significantly enhanced lysozyme activity in the serum of Nile tilapia. Among the groups, the GAF treatment exhibited the highest lysozyme activity, followed by AE, GE, and the control group, respectively. These findings indicate that both individual and combined herbal extracts effectively stimulate the non-specific immune system in fish, with the combined formulation demonstrating a clear synergistic effect. The significantly elevated lysozyme activity in the GAF group suggests a synergistic interaction between allicin from garlic and andrographolide from *A. paniculata*. This synergistic mechanism may be attributed to the immune-activating effects of garlic, which contains several biologically active sulfur compounds such as allicin, diallyl sulfide, and S-allyl cysteine. These compounds are known to stimulate various immune cells, including macrophages, neutrophils, and natural killer (NK) cells. Importantly, they have been shown to enhance lysozyme production in immune cells and strengthen host defense against bacterial infections [6]. This aligns with previous reports demonstrating garlic’s ability to upregulate lysozyme gene expression and protein synthesis in fish [[29], [30]]. Meanwhile, *A. paniculata* contains andrographolide, a diterpenoid lactone with well-documented anti-inflammatory and immunomodulatory properties. This compound has been shown to enhance macrophage activity and promote the production of antimicrobial substances such as nitric oxide (NO) and lysozyme [[26], [31]]. These effects are likely mediated through the activation of signaling pathways such as NF-κB and MAPK, which regulate gene expression involved in innate immune responses [[5], [32]]. Given that lysozyme is a key enzyme in the non-specific immune system, functioning to hydrolyze the peptidoglycan layer of bacterial cell walls, the significantly enhanced lysozyme activity observed in the GAF group implies improved disease resistance. Therefore, incorporating GAF into the diet could serve as an effective immunostimulant strategy to reduce disease susceptibility in high-density aquaculture systems.

The mechanism of action of these supplements were evaluated on the expression of inflammation-related genes in LPS -stimulated RAW 264.7 macrophage cells. The experimental results revealed that all three extracts significantly downregulated the expression of pro-inflammatory cytokine genes, including IL-1β, TNF-α, iNOS, and IL-6, compared to the LPS-treated control group. Among the treatments, AE exhibited the most pronounced anti-inflammatory effect, followed by the combined extract (GAF), both of which showed consistent and substantial inhibition across all gene targets. These findings are in line with previous reports by [32] and [26], which revealed that the bioactive compound andrographolide, the major constituent of *A. paniculata*, suppresses inflammation by inhibiting key signaling pathways, including NF-κB and MAPK, responsible for the transcription of cytokine genes. Although GE did not exert the same magnitude of suppression as AE, it still showed a notable anti-inflammatory effect, particularly in reducing the expression of IL-1β and TNF-α, which play crucial roles in initiating immune responses. The mechanism of garlic is believed to be related to its sulfur-containing compounds, such as allicin, S-allyl cysteine, and diallyl disulfide, which are known to inhibit nitric oxide production and reduce proinflammatory cytokine release in immune cells [23,25]. The GAF formulation, although not showing the highest inhibition in every cytokine marker, consistently downregulated all proinflammatory gene expressions. This suggests that GAF may help maintain immune balance without overly suppressing immune function, which is particularly beneficial for application in aquaculture during stress recovery or post-infection periods. The effects of GAF may result from synergistic interactions between the active compounds in AE and GE, or potentially from competitive effects that modulate their individual bioactivities. Further studies, including protein-level analyses such as ELISA or Western blot, are recommended to confirm these mechanisms at the post-transcriptional level. Additionally, treatment of RAW 264.7 cells with these extracts at concentrations ranging from 250 to 4000 µg/mL did not induce cytotoxicity. On the contrary, some concentrations, particularly in AGF, AE and GE groups, even enhanced cell viability. These observations support previous studies [8,6], which reported that andrographolide and allicin improve cellular stability and metabolic activity in immune cells without toxic effects. Moreover, the IL-6/IL-10 ratio, a widely used indicator of immune balance, was significantly reduced in all extract-treated groups compared to the LPS group. This finding implies the extracts’ potential to restore immune homeostasis by suppressing proinflammatory cytokines while maintaining anti-inflammatory responses within a functional range.

## Conclusion

The extracts of *Andrographis paniculata* (AE), *Allium sativum* (GE), and their combined formulation (GAF) exhibited promising potential for enhancing digestive function, stimulating immune responses, and modulating inflammation both at the organismal and cellular levels. Notably, the GAF formulation exhibited a well-balanced effect on both the digestive and immune systems. These results support the application of these herbal extracts as functional additives to reduce antibiotic usage, improve aquaculture efficiency, promote fish health, and minimize environmental impact which aligns with sustainable aquaculture principles.

## Funding

This research received funding by The Thailand Research Fund (Research and Researchers for Industries–RRI) Program (Project Code: PHD58I00069) and the Center of Excellence in Agricultural Innovation for Graduate Entrepreneur, Maejo University, Chiang Mai Province, Thailand.

## Institutional review board statement

The animal study protocols were approved by the Maejo University Animal Care and Use Committee, Chiang Mai, Thailand (Approval No. MACUC 010F/2568X).

## CRediT authorship contribution statement

**Mallika Supa-aksorn:** Writing – original draft, Supervision, Software, Methodology, Investigation, Formal analysis, Data curation. **Sudaporn Tongsiri:** Supervision, Resources, Methodology. **Jongkon Promya:** Supervision. **Chetsalit Hongnueng:** Visualization, Software, Data curation. **Doungporn Amornlerdpison:** Writing – review & editing, Validation, Supervision, Resources, Project administration, Methodology, Funding acquisition, Conceptualization.

## Declaration of competing interest

The authors declare that they have no known competing financial interests or personal relationships that could have appeared to influence the work reported in this paper.

## Data Availability

Data will be made available on request.

## Appendix Supplementary materials

Image, application 1

Image, application 2

Image, application 3
